# A late Paleocene probable metatherian (?deltatheroidan) survivor of the Cretaceous mass extinction

**DOI:** 10.1038/srep38547

**Published:** 2016-12-07

**Authors:** Xijun Ni, Qiang Li, Thomas A. Stidham, Lüzhou Li, Xiaoyu Lu, Jin Meng

**Affiliations:** 1 Key Laboratory of Vertebrate Evolution and Human Origins, Institute of Vertebrate Paleontology and Paleoanthropology, Chinese Academy of Sciences, 142 Xi Zhi Men Wai Street, Beijing, 100044, China; 2 CAS Center for Excellence in Tibetan Plateau Earth Sciences, Beijing, 100101, China; 3 University of Chinese Academy of Sciences, Beijing, 100049, China; 4 Division of Paleontology, American Museum of Natural History, Central Park West at the 79th Street, New York, NY 10024, USA.

## Abstract

Deltatheroidans are primitive metatherian mammals (relatives of marsupials), previously thought to have become extinct during the Cretaceous mass extinction. Here, we report a tiny new deltatheroidan mammal (*Gurbanodelta kara* gen. et sp. nov.) discovered at the South Gobi locality in China (Xinjiang Province) that is the first Cenozoic record of this clade and renders Deltatheroida a Lazarus taxon (with a new record 10 million years younger than their supposed extinction). The vertebrate fauna associated with *Gurbanodelta* is most similar to that from the slightly older late Paleocene Subeng locality in Inner Mongolia. The upper molars of *Gurbanodelta* exhibit a broad stylar shelf with one prominent cusp (stylocone), and a paracone that is sharp and significantly taller than the metacone. The lower molar tentatively assigned to *Gurbanodelta* has a very small talonid without an entoconid. This combination of these features is known only in deltatheroidans. Phylogenetic analysis places *Gurbanodelta* as the sister taxon of the North American latest Cretaceous *Nanocuris*. *Gurbanodelta* is the smallest-known deltatheroidan, and roughly the same size as the smallest living marsupial. It is likely that the *Gurbanodelta* lineage dispersed between Asia and North America as part of known intercontinental mammalian dispersals in the late Paleocene, or possibly earlier.

The Cretaceous-Paleogene (K-Pg) transition is marked by one of the largest mass extinctions, and its aftermath saw a reorganization of global ecosystems. One of the best-known post-Mesozoic changes is the rapid diversification of placental mammals, and that Cenozoic radiation is coincident with a dramatic decline among metatherians in the Northern Hemisphere, which also originated in the Mesozoic^1^,^2^,^3^,^4^. Deltatheroidans are basal metatherians (the clade including marsupials and all mammals more closely related to them than to placentals or monotremes) and known only from the Cretaceous of Asia and North America^1^,^2^,^5^,^6^,^7^,^8^,^9^. Given the published fossil record, it was thought that deltatheroidans became extinct at the end of the Cretaceous^1^,^9^ with the youngest Mesozoic records (*Nanocuris*) from the latest Cretaceous sediments of western North America^10^. However, we describe here a new deltatheroidan taxon from the late Paleocene of China. Morphological comparison and phylogenetic analysis suggest that this new species is more closely related to the North American latest Cretaceous *Nanocuris* than to other Asian deltatheroidans. This discovery is the first-known evidence demonstrating that deltatheroidans survived the Cretaceous mass extinction, and furthermore, that the group dispersed between North America and Asia late in their history.

In deltatheroidans, the last molariform deciduous premolar is not replaced in adulthood, and the tooth position is traditionally referred as M1/m1^1^,^2^,^8^,^11^. This morphology is shared with marsupials, but not placentals^11^. The upper molars of deltatheroidans are characterized by a broad stylar shelf, a small and mesiodistally compressed protocone, a particularly strong postmetacrista, small conules and stylar cusps, a paracone taller than the metacone, a high preprotocrista extending to buccal side and a short postprotocrista not extending buccally past the metacone. The lower molars have a trigonid that is much taller and broader than the talonid, a lingually positioned paraconid and a carnassial notch on the paracristid. The salient postmetacrista and paracristid form a postvallum/prevallid shearing mechanism, a feature adapted for a carnivorous diet. The preprotocrista and preparacrista form a surface for the double-rank prevallum/postvallid shearing mechanism. This combination of dental features supports the monophyly of Deltatheroida^1^,^9^,^12^,^13^.

## Geological background

**Figure 1:**
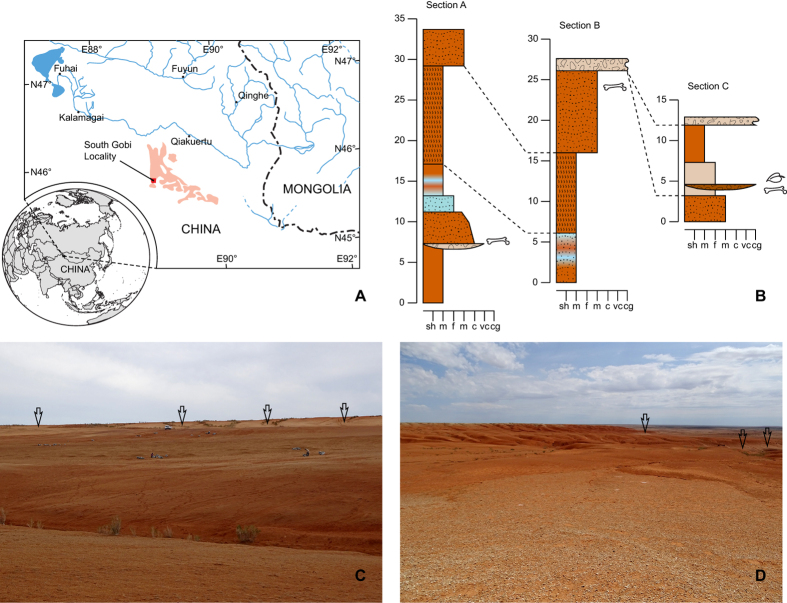
Geographic location and stratigraphic sections. (**A**) Map showing the location of the South Gobi locality in the Northeastern Junggar Basin in Xinjiang, China. The pale reddish area indicates the distribution of Cenozoic sediments. The global map is modified from https://en.wikipedia.org/wiki/File:Mongolia_(orthographic_projection).svg (under the Creative Commons Share Alike license: https://creativecommons.org/licenses/by-sa/3.0/deed.en). The zoomed-in map was produced by the first author based on satellite images in Google Earth (Version 7.1.5.1557, https://kh.google.com) and observations in the field by using Adobe Illustrator CS6 (Version 16, https://www.adobe.com). (**B**) Stratigraphic columns measured at the South Gobi locality. The late Paleocene fossil layer is indicated in the lower part of Section A. (**C**) Photo showing where the Section A was measured. (**D**) Photo showing where Sections B and C were measured. Black arrows indicate the gypsum-rich layer.

The new deltatheroidan fossils reported here were discovered in an unnamed early Cenozoic lithological unit at the South Gobi locality in the Gurbantunggut Desert in the Northeastern part of the Junggar Basin in Xinjiang Province, China (Fig. 1A). This unnamed unit is a set of sediments consisting of reddish-brown mudstones embedded with a few grayish-green or yellowish-gray fluvial sandstone beds or lenses (Fig. 1B). The upper part of the unit is a reddish-brown sandy mudstone bed containing abundant calcareous concretions. Below that bed is a thick reddish-brown mudstone containing a large amount of gypsum crystals, and the weathered surface of this layer is pale-reddish in color. The concretion-rich layer and the gypsum-rich layer are laterally extensive and can be traced transversely over tens of kilometers (Fig. 1C,D). Fossils were discovered in the sandstone lenses near the top of the whole section (Section C, Fig. 1B), the concretion-rich mudstone above the gypsum-rich layer (Section B, Fig. 1B), and the sandy mudstone with sandstone lenses below the gypsum-rich layer (Section A, Fig. 1A). The stratigraphically highest fossiliferous layer produced petrified wood and turtle carapace fragments. The middle fossiliferous layer (the concretion-rich mudstone layer), yielded only two rodent jaws, identified as *Advenimus hubeiensis* and *Tamquammys robustus*. Both species are typical of the early Eocene Bumbanian Asian Land Mammal Age (ALMA)^14^,^15^. The lowest layer produced the majority of fossils, including the new metatherian.

The non-mammalian vertebrates from the lowest fossiliferous layer include bone fragments of osteichthyans, amphibians, chelonians, squamates and crocodylians. There are many small fish specimens, and based on the size and morphological differences among the vertebrae, those specimens likely represent a minimum of two species. The frog specimens derive from relatively small sized frogs, and likely are from a minimum of two species. There are several carapace fragments from a relatively large trionychid turtle, and the morphology of those specimens is consistent with a single taxon. The lizard material is composed of scales, jaw fragments, and a humerus fragment. There are dentaries with acrodont and non-acrodont dentitions, and the tooth morphologies on those specimens vary, suggesting the occurrence of up to five species. The morphology of the lizard scales and some jaw fragments suggests that they derive from a non-glyptosaurine anguioid lizard. Anguioids are well known in the late Cretaceous and Paleogene of Asia and North America^16^. The morphology of the anguioid specimens from the South Gobi locality is almost identical to the unnamed anguioid from the late Paleocene Nomogen Formation at the Subeng locality in Erlian Basin of Inner Mongolia, China^17^. The acrodont jaw fragments, including one specimen with tricuspid teeth, suggest that at least one species represents an iguanian. The crocodylian material comprises several fragments of large osteoderms and several small laterally compressed teeth. The osteoderms are consistent with that of dyrosaurids, and would appear to be from individuals much larger than those that would likely have produced the teeth (that are only a few millimeters across). Overall, this non-mammalian vertebrate fauna, along with abundant gyrogonites (from charophyte algae) support a paleoenvironment with abundant aquatic habitats.

**Figure 2:**
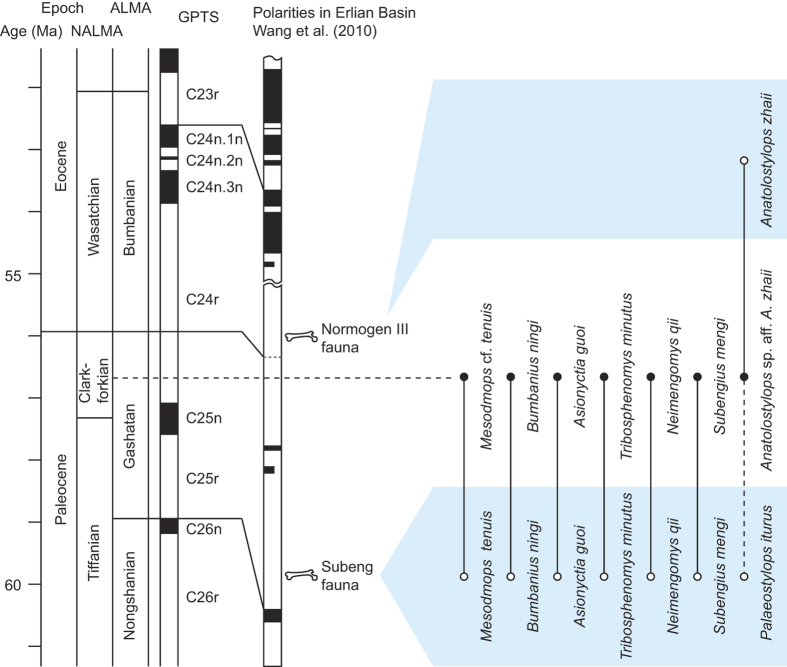
Mammalian fossils from South Gobi locality correlated with the Subeng fauna and Nomogen III fauna from the Erlian Basin in Inner Mongolia, China. The age of South Gobi fossil assemblage is estimated as late Gashatan ALMA, roughly equivalent to the Clarkforkian NALMA.

The matrix collected from the sandstone lens embedded in the lowest fossiliferous layer was screen-washed, and it produced small mammalian fossil remains representing eight species, including the deltatheroidan specimens reported here (Fig. 2). Among these fossils are species of insectivorans (*Bumbanius ningi* and *Asionyctia guoi*), Glires (*Tribosphenomys minutus* and *Neimengomys qii*), plesiadapiforms (*Subengius mengi*) and multituberculates (*Mesodmops* cf. *tenui*s), species that were previously only known from the Nomogen Formation at the Subeng locality (Fig. 2)^18^,^19^. The arctostylopid from the South Gobi locality is much larger than *Palaeostylops iturus* from the Subeng locality, and approaches the size of *Anatolostylops zhaii* from the early Eocene Nomogen III Fauna of the Bumbanian ALMA (Fig. 2)^20^,^21^.

Biostratigraphic and magnetostratigraphic data suggest that the Paleocene-Eocene Boundary in the Erlian Basin lies in or just below the “*Gomphos*” bed^20^,^22^,^23^. The mammalian fossils from the “*Gomphos*” bed comprise the Nomogen III Fauna of the Bumbanian ALMA, dominated by specimens of *Gomphos*. No specimens of *Gomphos* have been found at the South Gobi locality. The age of mammalian fauna from the Subeng locality is early Gashatan ALMA (late Paleocene), roughly equivalent to the late Tiffanian North American Land Mammal Age (NALMA)^20^,^22^,^23^. The presence of *Anatolostylops* sp. aff. *A. zhaii* at the South Gobi locality indicates a somewhat younger age than that of the Subeng Fauna, and the absence of the common *Gomphos* fossil from the South Gobi locality may suggest an older age than the Nomogen III Fauna. The Paleocene multituberculate *Lambdopsalis bulla* is the most common element in the Nomogen III Fauna, and is absent at the South Gobi locality. Given those biostratigraphic data, we correlate the mammalian fauna from the South Gobi locality to the late Gashatan ALMA, roughly equivalent to the Clarkforkian NALMA. Faunas of this age were previously unknown in Asia, and this locality and its fauna are approximately 10 million years younger than the Cretaceous mass extinction.

## Result

Class Mammalia Linnaeus, 1758

Subclass Tribosphenida McKenna, 1975

Infraclass Metatheria Huxley, 1880

Order Deltatheroida Kielan-Jaworowska, 1982

Family Deltatheridiidae Gregory & Simpson, 1926

*Gurbanodelta kara* gen. et sp. nov.

### Etymology

The genus name is derived from the Gurbantunggut Desert where the holotype was found and delta, a common component of deltatheroidan names. The specific epithet is from ‘kara,’ meaning black in the local Kazakh language.

### Holotype

**Figure 3:**
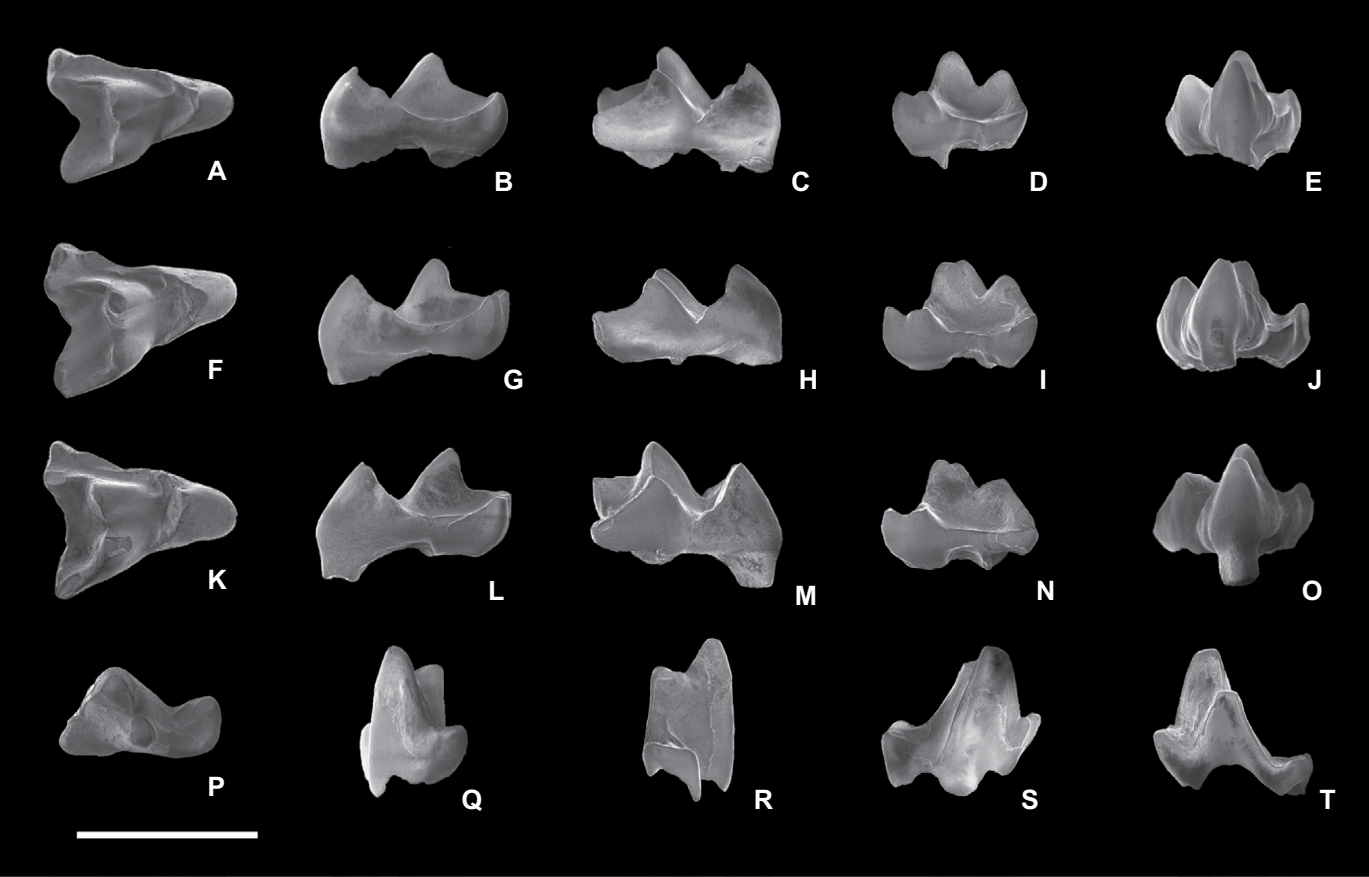
Dentition of *Gurbanodelta kara* gen. et sp. nov. (**A–E**) Right M2, IVPP V 22801. (**F–J**) Right M2, IVPP V 22802, holotype. (**K–O**) Right M3, IVPP V 22803. (**P–T**) Right m1, IVPP V 22804. From left to right, the images are in occlusal, mesial, distal, buccal and lingual views, respectively. Scale bar indicates 1 mm.

Institute of Vertebrate Paleontology and Paleoanthropology (IVPP), V 22802 (Fig. 3F–J), a right M2.

### Hypodigm

IVPP V 22801, a right M2; IVPP V 22803, a right M3; IVPP V 22804, a right m1.

### Locality and horizon

South Gobi mammalian fossil locality, Kalabulegen Town, Xinjiang Province, China (Fig. 1), late Gashatan ALMA in the late Paleocene.

### Diagnosis

Smallest known deltatheroidan (Table 1), much smaller than all other deltatheroidans. Differs from all other deltatheroidans in having a stylar shelf narrower than the half of the width of the tooth, and a paraconid smaller and lower than the metaconid on m1. Differs from all deltatheroidans (except *Atokatheridium*) in lacking a buccal cingulum on the upper molars, and having a fully developed postmetacrista on M3. Differs from all deltatheroidans (except *Tsagandelta* and *Deltatheroides*) in having a narrow talonid that occupies half of the molar’s width, and differs from *Tsagandelta*, *Sulestes* and *Deltatheroides* in lacking a sharp mesial keel below the paraconid.

### Description

The upper teeth (identified as M2–3) all have a triangular crown with three trenchant, buccally leaning cusps (the protocone, paracone and metacone). The mesial border of the tooth is straight. The buccal border is concave between the paracone and metacone (i.e., the ectoflexus is deep). Distally, the border between the protocone and metacone also has a shallow indentation. The protocone is a trenchant cusp, and the cusp is quite small relative to the tooth size, as in all deltatheroidans. The cusp is mesiodistally very compressed and buccolingually quite narrow. Both the preprotocrista and postprotocrista of the protocone are very sharp and strong. The former extends all the way to the buccal side and terminates at the large parastyle. The latter is very short, and it extends to the lingual side of the metacone only, not passing the base of the metacone. The pre- and postprotocristae connect to each other at the tip of the protocone at a narrow angle. The buccal surface of the protocone (bordered by those two strong ridges) is therefore narrow and concave. The lingual surface of the protocone is very narrow and forms a round ridge. There is no cingulum developed on the mesial, lingual or distal surfaces of the protocone. The paracone is smaller, but taller than the protocone. It has a convex and ridged lingual surface, and has a narrow and concave buccal surface. The preparacrista of the paracone is very long. It extends mesiobuccally for a short distance, and makes a sharp turn to the buccal side, becoming parallel to the buccal part of the preprotocrista. Eventually, the preparacrista terminates at the stylocone, distal to the parastyle. The postparacrista is short and low, and extends distally to meet the premetacrista of the metacone. The metacone is much smaller and lower than the paracone. The premetacrista of the metacone is short and low, and has the same form as the postparacrista. The postmetacrista is very strong, with its buccal part even longer and higher than the preparacrista; this feature is related to the postvallum-prevallid shearing mechanism.

The protocone, paracone and metacone enclose a very narrow but deep trigon basin. Buccal to the paracone and metacone, the tooth has a very broad stylar shelf. Buccolingually, the shelf occupies more than one-third of the tooth’s total width. The bottom of the stylar shelf is very smooth and bowl shaped, and its buccal edge has a poorly developed cingulum.

The conules and styles of the upper molars are moderately developed. Both the paraconule and metaconule are present as small swollen nodules on the preprotocrista and postprotocrista, and they do not project more ventrally than the two cristae. The paraconule is larger than the metaconule. A weak postparaconule crista extends to the base of the paracone. The metaconule does not have cristae. On the buccal side, only the parastyle and stylocone are relatively well developed, and they are present as twinned cusps located at the mesiobuccal corner of the tooth, barely projecting above the cristae connected to them. Near the parastyle, the buccal part of the preprotocrista has a tiny swollen nodule that forms a small cusp-like structure. Two similar tiny nodules also are present on the buccal border of the stylar shelf, and may be equivalent to the stylar cusps in other metatherians.

The three upper molars are quite similar to each other. IVPP V 22801 and V 22802 are identified as M2s, because the protocones of the two teeth are mesiodistally symmetrical. The distobuccal ends of the postmetacristae in the two teeth are elevated. In buccal view, the elevated part looks like a metastyle. V 22801 is smaller than V 22802. The twinned parastyle and stylocone of V 22801 are very close to each other, whereas the two cusps in V 22802 are well separated. V 22803 is slightly larger than the M2s and identified as an M3. The protocone of this M3 is mesially tilted, and as a result, its distal surface is slightly bigger than its mesial surface. The ectoflexus of the buccal tooth border of M3 is shallower than that of M2. The twinned parastyle and stylocone of M3 are well separated as in M2 (V 22802, but not V22801). The postmetacrista of M3 is quite straight, and its distobuccal end is not elevated.

The tentative attribution of the lower molar (IVPP V 22804) to *Gurbanodelta kara* is based on its very small size, and its combination of a triangular trigonid, well developed and widely separated paraconid and metaconid, trenchant protoconid, and long but narrow talonid. The eutherian insectivores from the same locality, such as *Bumbanius ningi* and *Asionyctia guoi*, are much larger than *G. kara*. The molars of these taxa have a more fully developed talonid with a large hypoconid and entoconid, and a broad talonid basin. The premolars of *A. guoi* and similar eutherian mammals are roughly similar to V 22804 in size, but the premolars of these mammals have a much weaker paraconid and metaconid, and a much shorter talonid than V 22804. Some eulipotyphlan placentals, such as *Plagioctenodon*, have molariform premolars. In these eutherians, the paraconid and metaconid of p4 are quite large, but differ from V 22804, with the metaconid more mesially positioned, and the paraconid and metaconid closer to each other. The carnassial notch between the protoconid and metaconid is absent in those eulipotyphlans. The p4 talonid is proportionally wider, and the difference in height between the trigonid and talonid is lower than in V 22084 and the molars of other deltatheroidans. In some basal marsupialiformes metatherians (= “traditional” definition of Marsupialia, e.g. in ref. 1), such as *Alphadon* and *Peradectes*, the trigonid of the lower molars has large, well-separated paraconids and metaconids, and notched shearing crests between the paraconid and protoconid and between the protoconid and metaconid. However, the talonids in these marsupialiform metatherians are proportionally much broader. The premolars in *Alphadon* or *Peradectes*-like marsupialiform are buccolingually narrow, with very weak paraconids and metaconids.

As a molar of a deltatheroidan, the small size, very open trigonid and low paraconid of V 22804 suggests that this tooth probably is an m1. The trigonid of the tooth is very tall. The protoconid is shaped like a triangular pyramid. The paraconid and metaconid are conical in shape, and all located near the lingual border of the tooth. The paraconid is smaller and lower than the metaconid. The enamel of the paraconid is chipped off; as a result, the cusp appears smaller than it would have been when intact. Both the paraconid and metaconid are much lower than the protoconid. The paraconid is widely separated from the metaconid, leaving the lingual side of the trigonid completely open. The distolingual side of the paraconid and the mesiolingual side of the metaconid are smooth, and no ridge connects the two cusps. Two blunt crests (the paracristid and protocristid) run down from the tip of the protoconid and connect to the paraconid and metaconid, respectively. The paracristid, linking the protoconid and paraconid, is long and straight. In mesiobuccal view, the paracristid is shaped like a checkmark. At the bottom of the checkmark, a rudimentary carnassial notch is developed (a lower molar feature related to the postvallum/prevallid shearing mechanism). The protocristid is short and has a shallow carnassial-notch-like structure. Along the distolingual margin of the metaconid, a weak but long distal metacristid is present. This ridge extends distally and continues to the lingual margin of the talonid.

The talonid of the lower molar is much narrower, shorter and lower than the trigonid. The hypoconid is the dominant cusp, and it barely projects more dorsally than the very short cristid obliqua. The cristid obliqua is low and weak. It extends from the mesial side of the hypoconid to connect to the distal wall of the metaconid. A tiny swollen nodule is developed on the lingual end of the hypocristid, and it can be interpreted as the rudimentary hypoconulid. The talonid basin is small and shallow with its lingual side is completely open. Many non-marsupial metatherians have mesial cingulid cusps at the base of the paraconid and protoconid, known as cuspid e and f. The enamel on the mesial side of V 22804 is broken, but the remaining dentine is smooth, and so it is unlikely that a cingulid and cingulid cusp were present.

The hypertrophy of postvallum-prevallid shearing in deltatheroidans results in a paraconid that is taller and larger than metaconid. A deep carnassial notch is always present on the paracristid. The talonid in deltatheroidans is small, but the hypoconid and hypoconulid are usually prominent. The lower molar (V 22804) referred to *Gurbanodelta*, has a large paraconid, but it is lower than the metaconid. The carnassial notch on the paracristid of this tooth is very shallow. The talonid is proportionally as small as those unequivocal deltatheroidans, but with a less projecting hypoconid and hypoconulid.

## Discussion

The tiny mammal *Gurbanodelta kara* reported here exhibits many characters diagnostic of deltatheroidan metatherians, and they strongly support the placement of *Gurbanodelta* within Deltatheroida, despite its Cenozoic late Paleocene age. The combination of the broad stylar with only one prominent stylar cusp (stylocone), a mesiodistally compressed protocone, poorly developed paraconule and metaconule, a strong and buccally extended preprotocrista combined with a very short postprotocrista, a sharp paracone significantly taller than the metacone, a long and strong postmetacrista, a very small lower molar talonid and an absent entoconid is not known in any other mammals other than deltatheroidans. A few Mesozoic and Cenozoic mammals share some of the characters mentioned above, but not all in combination. For example, the early Eocene *Didelphodus* from North America and Europe is thought to be the most plesiomorphic placental mammal^2^,^24^, and it retains a relatively broad stylar shelf, salient postmetacrista, a paracone higher than the metacone and talonid much lower than the trigonid. However, *Didelphodus* has a relatively large protocone with a well-developed paraconule and metacone. The buccal part of its preprotocista is low and narrow, and its postprotocrista is long and extends to the distal side of the metacone. Furthermore, its lower molar has a large talonid with a well-developed entoconid. Basal marsupialiform metatherians, such as *Alphadon* and *Peradectes*, share a broad stylar shelf and strong preparacrista and postmetacrista with *Gurbanodelta* and other deltatheroidans. These basal marsupialiform metatherians differ from deltatheroidans in having a paracone that is usually lower than the metacone, a non-compressed protocone, and a large talonid (both buccalingally and mesiodistally) on the lower molars.

In early eutherians, the postprotocrista of the upper molars extends buccally past the base of the metacone, creating a so-called “double-rank postvallum/prevallid shearing” mechanism with the postmetacrista^12^,^25^,^26^,^27^. In deltatheroidans, the postprotocrista is very short, and the postmetaconule crista and postcingulum are absent. The postvallum/prevallid shearing mechanism is formed only by the salient postmetacrista^12^,^25^,^26^,^27^. This shearing mechanism is convergently emphasized in many phylogenetically distant carnivorous mammals, such as deltatheroidans, borhyaenoids, stagodontids, dasyuroids, creodonts, carnivorans, and *Prionogale*^28^. However, deltatheroidans are strikingly different from these other carnivorous mammals in having a very broad stylar shelf and a paracone saliently larger and taller than the metacone. As in some basal therians (and differing from eutherians and marsupial metatherians), the preprotocrista in deltatheroidans extends buccally and forms the double-rank prevallum/postvallid shearing^12^,^25^,^26^,^27^. The molars of *Gurbanodelta* clearly show the double-rank prevallum/postvallid shearing and the strong postvallum/prevallid shearing. However, the postvallum/prevallid shearing in *Gurbanodelta* is not very strongly developed, because the postmetacrista-paracristid is not greatly stronger than the preparacrista-protocristid. This situation is unlike other deltatheroidans except *Nanocuris*, probably being related to the very small size of the teeth of *Gurbanodelta*, and a transition to a more insectivorous lifestyle from a more carnivorous ancestry.

**Figure 4:**
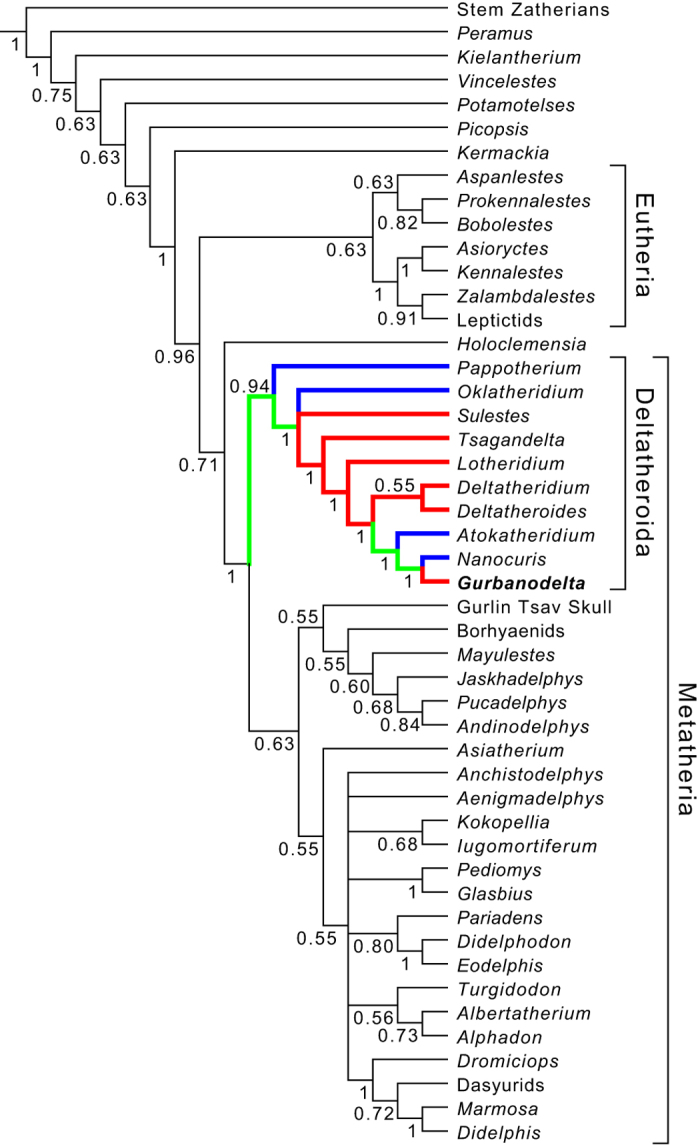
Majority rule consensus tree derived from 97 equally-parsimonious trees (569 steps) resulting from the deltatheroidan-focused data matrix. The numbers at the nodes indicate the consensus percentage. The strict consensus of those trees does not fully resolve the positions for most of the placental and marsupial mammals, but the relationships among deltatheroidans are well supported. The geographic distribution of deltatheroidans was mapped on the phylogenetic tree and the ancestral states were reconstructed with parsimony criteria with Mesquite 3.03 (ref. 48). Red clades represent Asian origin, and blue clades represent North American origin. Green clades indicate equally-parsimonious or ambiguous Asian or North American origins.

Our identification of *Gurbanodelta* as a deltatheroidan metatherian is based on detailed morphological comparisons (Supplementary Information), but is also by phylogenetic analyses (Fig. 4 and S-Fig. 1 in Supplementary Information). Parsimony analyses based on the dataset of Rougier *et al*.^9^, which includes all the deltatheroidan genera, reveal that *Gurbanodelta* is the sister species of the North American latest Cretaceous *Nanocuris*, and an analysis using a published broader sampling of therian mammals^27^ also places our new taxon among delatheroidans at the base of Metatheria, and not among any other clade of mammals (Supplementary Information). The two deltatheroidan taxa are joined by *Atokatheridium* to form a monophyletic group, with Asian Cretaceous deltatheroidans forming successive stems (Fig. 4). Given the inferred ~10 million ghost lineage leading to *Gurbanodelta* from its North American sister *Nanocuris*, the occurrence of *Gurbanodelta* in the late Paleocene of China (after the group’s supposed extinction at the end of the Cretaceous) would render it a Lazarus taxon^29^.

*Gurbanodelta* and its phylogenetic position within Deltatheroida demonstrates that at least one lineage of this metatherian group survived the K-Pg mass extinction. The body mass of *Gurbanodelta* was very small. Its molar area (less than 1 mm^2^) is only about 1/20th of that in *Nanocuris*. By using the predictive formulae from regression analyses of tooth area and body mass in extant, dentally conservative marsupials^30^, the body mass of *Gurbanodelta* is estimated at 4.3 g, suggesting that *Gurbanodelta* is roughly the same size as the long-tailed planigale (*Planigale ingrami*), the smallest living marsupial, and one of the smallest of all mammals^31^. The inferred diet of deltatheroidans, focusing on insectivory/carnivory, and their small body mass may explain their survival into the Cenozoic. It has been suggested that smaller-bodied, less specialized carnivores and animal-dominated omnivores had a better chance of survival^32^. Since *Nanocuris* was a part of the latest Cretaceous mammalian fauna in North America^32^, it seems highly probable that deltatheroidans survived there locally, but have yet to be sampled. A deltatheroidan similar to *Atokatheridium* and *Gurbanodelta* may be lurking somewhere undiscovered in North American Paleogene sediments. Further effort to examine small-sized mammalian teeth in the early Cenozoic likely will produce a fuller record of deltatheroidans, and potentially individuals of the *Gurbanodelta* lineage.

The K-Pg event is the most recent of the five largest mass extinctions^33^,^34^,^35^. Although most of the Mesozoic mammals became extinct by the end of Cretaceous, a few Mesozoic lineages are now known to have survived into the Cenozoic^3^,^32^,^36^. Those Mesozoic survivors on the northern continents include the stem therian multituberculates, a stem marsupial lineage leading to *Peradectes*, stem placental cimolestids, placental lineages leading to archaic ungulates (*Protungulatum*), a member of Zhelestidae and now a deltatheroidan^3^,^37^. On the southern continents, Mesozoic lineages, such as the australosphenidan monotremes, the gondawanatherians and the meridiolestidans are known to have been coeval with the Cenozoic eutherians and metatherians for millions of years^36^. The presence of those diverse Mesozoic survivors at the beginning of Cenozoic suggests that those archaic mammals probably still occupied a broad spectrum of ecological niches.

When the geographic distribution of the deltatheroidans is mapped onto the phylogenetic tree and ancestral states are reconstructed using maximum parsimony, the results indicate that an Asian or a North American origin for the deltatheroidan lineage represented by *Gurbanodelta* are equally parsimonious (Fig. 4). *Gurbanodelta* from the late Paleocene creates a gap in the deltatheroidan fossil record of nearly 10 million years, and a gap in the Asian fossil record of the group of over 20 million years. Given the temporal distribution of the youngest fossils (late Paleocene and Late Cretaceous) and their hypothesized phylogenetic relationships, it seems more likely that the *Gurbanodelta* lineage dispersed from North America to Asia (in the latest Cretaceous or Paleocene), or that the *Nanocuris* lineage dispersed to North America (with a cryptic *Gurbanodelta* lineage present in Asia) in the Cretaceous. The dispersal of the Cenozoic deltatheroidan lineage, from North American to Asia, or from Asia to North America in the Mesozoic, occurred prior to the well-known mass interchange of mammals and other taxa at the Paleocene-Eocene Boundary^38^,^39^,^40^,^41^,^42^. This metatherian dispersal, if Paleocene rather than Cretaceous in age, may have been penecontemporaneous with known Asian-North American intercontinental dispersals of the middle to late Paleocene that included the movement of tillodonts, rodents, plesiadapiforms, and other vertebrates^38^,^39^,^41^,^42^,^43^.

## Methods

### Measurements

Specimens were measured under the ZEN Pro 2012 system stored with a Zeiss stereo-microscope (Discovery V20), and were calibrated from the caliper. The results are listed in Table 1.

### Phylogenetic analysis

We added *Gurbanodelta kara* to the dataset of Luo *et al*.^27^ to examine the systematic position of *Gurbanodelta* relative to Deltatheroida within a broader mammalian sample (Supplementary Information), and the recent dataset of Rougier *et al*.^9^ to examine the phylogenetic relationships between *Gurbanodelta* and other deltatheroidans more specifically. The deltatheroidan matrix is based on Rougier *et al*.^44^ and recently revised by Averianov *et al*.^45^, Wilson and Riedel^10^, and Rougier *et al*.^9^. In total, 53 taxa were scored for 156 characters. We followed the suggestion of Rougier *et al*.^9^ and excluded the taxa that have an uncertain tooth locus (i.e., *Slaughteria*) or that are known from a single tooth (i.e., *Aegialodon*, *Comanchea*, *Trinititherium*, *Falepetrus*, and *Zygiocuspis*) from the analysis. Stem zatherians, as represented by a compound taxon of *Amphitherium* plus dryolestids, were utilized as the outgroup.

Parsimony analysis was undertaken using TNT (Tree analysis using New Technology), a parsimony analysis program subsidized by the Willi Hennig Society^46^. The parsimony searching strategy of Ni *et al*.^47^ was used. We ran multiple replications, using sectorial searches, drifting, ratchet and fusing combined. Random sectorial search, constraint sectorial search and exclusive sectorial search were set on. Ten cycles of tree drifting, 10 cycles of ratchet and 10 cycles of tree fusing were performed in the search. Default parameter settings for random sectorial search, constraint sectorial search, exclusive sectorial search, tree drifting, ratchet and fusing were used. The search level was set as 10 for 103 (Luo’s dataset)^27^ and 47 (Rougier’s dataset)^9^ taxa. Optimal scores were searched with 10000 replications. Eleven characters are set as ordered. All characters have equal weight.

## Additional Information

**How to cite this article**: Ni, X. *et al*. A late Paleocene probable metatherian (?deltatheroidan) survivor of the Cretaceous mass extinction. *Sci. Rep.* **6**, 38547; doi: 10.1038/srep38547 (2016).

**Publisher's note:** Springer Nature remains neutral with regard to jurisdictional claims in published maps and institutional affiliations.

## Supplementary Material

Supplementary Information

Supplementary Dataset 1

Supplementary Dataset 2

## Figures and Tables

**Table 1:** Measurements of the specimens.

Specimen number	Tooth	Length (mm)	Mesial width (mm)	Distal width (mm)
IVPP V 22801	M2	0.75	1.05	1.08
IVPP V 22802	M2	0.86	1.1	1.1
IVPP V 22803	M3	0.88	1.12	1.12
IVPP V 22804	m1	0.9	0.6	0.4
