# Supplementary material for: A late Paleocene probable metatherian (?deltatheroidan) survivor of the Cretaceous mass extinction
Source: Sci Rep. 2016 Dec 7;6:38547. doi: 10.1038/srep38547 (PMC5141426; doi:10.1038/srep38547)
Supplement: Supplementary Information [file srep38547-s1.pdf]

Supplementary Information for

A late Paleocene probablemetatherian (?deltatheroidan) survivor of the Cretaceous mass extinction

Xijun Ni[1,2,3], Qiang Li[1,2,3], Thomas A. Stidham[1,3], Lüzhou Li[1,3], Xiaoyu Lu[1,3], Jin Meng[4]

1. Key Laboratory of Vertebrate Evolution and Human Origins, Institute of Vertebrate Paleontology and Paleoanthropology, Chinese Academy of Sciences, 142 Xi Zhi Men Wai Street, Beijing, 100044, China
2. CAS Center for Excellence in Tibetan Plateau Earth Sciences, Beijing, 100101, China
3. University of Chinese Academy of Sciences, Beijing 100049, China
4. Division of Paleontology, American Museum of Natural History, Central Park West at the 79th Street, New York, NY10024

Correspondence to: nixijun@ivpp.ac.cn

This file includes:

Comparisons
Supplementary references

Other Supplementary Materials for this paper includes the following:

Supplementary Datasets as in TNT format:
Rougier_et_al_2015_plus_ni_et_al.doc
Luo_mbank_X1599_plus_ni_et_al.doc

Comparisons

The earliest known deltatheroidans include the early Cretaceous (Aptian-Albian) *Atokatheridium boreni*, *Oklatheridium szalayi*, *Oklatheridium minax*, and *Oklatheridium* sp. from the Antlers Formation in the United States (Texas and Oklahoma) [1-3]. The Aptian-Albian *Pappotheridium pattersoni* from the upper part of the Antlers Formation in north central Texas was traditionally identified as "Theria of Metatherian-Eutherian Grade" [4] and more recently has been considered as a "tribothere" because it exhibits a primitive tribosphenic form lacking the diagnostic traits of Metatheria and Eutheria [5]. However, some recent phylogenetic analyses (including our own, see below) place *Pappotheridium* as the most basal deltatheroidan [3,6], but some others have suggested that *Pappotheridium* is not a metatherian [7,8]. The youngest known records of North American deltatheroidans are *Nanocuris improvida* and an unnamed species that probably belongs in *Nanocuris* [7]. Those taxa occur in the Lancian NALMA (Maastrichtian, latest Cretaceous) of the Lance Formation in the USA, and the Frenchman and Scollard Formations in Canada [7]. The oldest records of deltatheroidans in Asia are from the late Cretaceous (Turonian), and are represented by *Sulestes karakshi* from the Bissekty Formation in Uzbekistan [9]. The best-known deltatheroid genus, *Deltatheriudium* (composed of two species, *D. pretrituberculare* and *D. nessovi*) is known from the Late Cretaceous (Campanian) of Mongolia (Djadokhta Formation, the "Ukhaa Tolgod Beds," the Baruungoyot Formation and the Red Beds of Hermiin Tsav in the Gobi Desert), China (the Bayn Mandahu Formation in Inner Mongolia) and Kazakhstan (Darbasa Formation) [10]. The relatively poorly known *Deltatheroides cretacicus* (also from the Djadokhta Formation in Mongolia) [10] was contemporaneous with *Deltatheriudium*. The recently reported *Tsagandelta dashzevegi* (from the lower levels of the Baynshiree Formation in eastern Mongolia) has an uncertain age, ranging from the Cenomanian to Campanian [6]. Another recently named species, *Lotheridium mengi*, was discovered in the late Cretaceous Qiupa Formation in Henan Province, China [11], but the exact locality and age of this species are not known. The youngest previously published specimens of delatheroidans from Asia were older than those from North America (Campanian vs. latest Maastrichtian), but *Gurbanodelta* demonstrates that there is a missing fossil record from either, or both, North America and Asia.

Pappotheridium and *Gurbanodelta* share some interesting similarities, such as strong preparacrista coupled with an elevated and buccally expanded preprotocrista, a sharp stylocone, a long postmetacrista without a carnassial notch and an unreduced metacone and postmetacrista lobe on M3. However, the two taxa also differ distinctively from each other in many features. The protocone of *Pappotheridium* is buccolingually broad, and proportionally broader than in *Gurbanodelta*. The postprotocrista in *Pappotheridium* is more buccally expanded than the state in *Gurbanodelta*. In *Pappotheridium*, the postprotocrista extends to the distolingual side of the metacone, and in *Gurbanodelta*, the postprotocrista terminates at the lingual side of the metacone. Associated with the relatively large protocone, *Pappotheridium* has a better-developed

talonid (as compared to *Gurbanodelta*) with well-differentiated hypoconids, hypoconulids and entoconids on the lower molars.

Deltatheridium and *Deltatheroides* are closely related taxa and their morphology is well known [12,13]. In comparison to the smaller *Gurbanodelta*, both *Deltatheridium* and *Deltatheroides* have proportionally broader buccal stylar shelves that occupy more than half of the tooth's width. The stylar shelf in *Gurbanodelta* occupies about one-third of the tooth's width. The ectoflexus of *Deltatheridium* and *Deltatheroides* are very deep, and the depth increases from M1 to M3. The ectoflexus of *Gurbanodelta* is moderately deep, and it becomes shallower from M2 to M3. The paracone and metacone of *Deltatheridium* and *Deltatheroides* closely approach each other, and their bases almost fuse together. Both cusps are slender. The paracone is only slightly taller than the metacone. The bases of the paracone and metacone are not completely fused together in *Gurbanodelta*, but they approach each other. The paracone and metacone are trenchant in shape, with the former much higher than the latter. The postmetacrista of M2 in *Deltatheridium* and *Deltatheroides* is very long and strong. A small notch is present on this crista, making it like a carnassial shearing blade. The postmetacrista of M2 in *Gurbanodelta* also is very strong, but those teeth lack such a notch. Relative to the preparacrista, the postmetacrista of M3 is reduced in *Deltatheroides*, and very reduced in *Deltatheridium*. In *Gurbanodelta*, the postmetacrista of M3 is as long as the preparacrista. The m1 (known in *Deltatheridium* but not in *Deltatheroides*) has a paraconid higher and larger than the metaconid. The hypoconid of the tooth is quite projecting. The m1 of *Gurbanodelta* has a paraconid lower and smaller than metaconid. Its hypoconid is low and small, and barely projects above the talonid. The recently described *Lotheridium* is very similar to *Deltatheridium* and *Deltatheroides*. The differences present between *Gurbanodelta* and *Deltatheridium* (plus *Deltatheroides*) are the same differences between *Gurbanodelta* and *Lotheridium*.

Sulestes has very broad stylar shelves on the upper molars, very strong postmetacristae with carnassial notch-like structures on M2 and a reduced metacone and postmetacrista lobe on M3 (similar to *Deltatheridium* and *Deltatheroides* but different from *Gurbanodelta*) [9]. The protocone of *Sulestes* is large and bears strong paraconule and metaconule. For a deltatheroidan, this morphology is quite unique. The ectoflexus of *Sulestes* is relatively shallow and broad, and the width of the stylar shelves decreases from M1 to M3. These two features are similar to *Gurbanodelta*. The m1 of *Sulestes* has a very large paraconid, larger and taller than the metaconid. A deep carnassial notch is present on the paracristid. Corresponding to the broad protocone, the talonid in *Sulestes* is also relatively large. The hypoconid and hypoconulid are prominent, and a rudimentary entoconid is always present. These lower molar characters are in a sharp contrast to those of *Gurbanodelta*.

Oklatheridium is a small deltatheroidan, but still larger than *Gurbanodelta.* The protocone in this species is relatively better developed than in *Gurbanodelta*. Its trigon basin is broader, and the conules are larger than those of *Gurbanodelta*. Its preprotocrista is relatively low. It extends to the buccal side past the mesial side of the paracone, but it is not elevated and closely approaches the base of the paracone. In *Gurbanodelta*, the

preprotocrista is elevated and well separated from the paracone. The relatively weak preprotocrista in *Oklatheridium* likely is coupled with more emphasis on the postvallum/ prevallid shearing than in *Gurbanodelta*. The postmetacrista of M2 in *Oklatheridium* is long and bear deep carnassial notches. The ectoflexus of the M2 is much deeper than the state in *Gurbanodelta*. The M3 of *Oklatheridium* has a reduced metacone lobe with very short postmetacrista, similar to that in *Deltatheridium* and *Deltatheroides*, but different from *Gurbanodelta*. The lower molar of *Oklatheridium* has a paraconid slightly larger and higher than the metaconid. Small cuspids e and f are present. The talonid is better developed than in other deltatheroidans, and both the hypoconulid and entoconid are present. These features of *Oklatheridium* are very different from the m1 of *Gurbanodelta*.

Similar to *Gurbanodelta*, the slightly larger *Atokatheridium* has a moderately broad stylar shelf, a mesiodistally compressed protocone, well-separated paracone and metacone, salient buccal expansion of preprotocrista, a shallow ectoflexus and a well-developed postmetacrista on M3. In both taxa, the postmetacrista lacks a carnassial notch. This absence may be related to their small body size and a lessened reliance on the postvallum/prevallid shearing. The parastyle and the stylocone in *Atokatheridium* are not twinned. The parastyle is more lingually positioned relative to the stylocone, and lower than the stylocone. The stylocone itself is quite blunt. The buccal part of the paracrista is low, and weakly connected to the stylocone. In *Gurbanodelta*, the parastyle and stylocone are twinned cusps. Both are buccally positioned, and are similar in height and size. The stylocone is conical. The paracrista extends buccally and connects the stylocone with a high ridge. The protocone of *Atokatheridium* is buccolingually broad, and proportionally wider than that of *Gurbanodelta*. The m1 of *Atokatheridium* has a paraconid slightly smaller than the metaconid, but the two cusps are similar in height. In *Gurbanodelta*, the paraconid is much lower than the metaconid.

The larger upper molars of *Nanocuris* have a proportionally longer crown outline than in *Gurbanodelta*. In *Nanocuris*, the protocone is relatively smaller. In mesial or distal view, it is significantly lower than the paracone and metacone. In *Gurbanodelta*, the protocone is slightly lower than the paracone and metacone. The paracone of *Nanocuris* has a rounded lingual border. In contrast, the lingual side of the paracone of *Gurbanodelta* forms a blunt ridge. The preparacrista of *Nanocuris* is relatively weak, and much shorter than the strongly distobuccally expanded postmetacrista. In *Gurbanodelta*, the two cristae are almost equally developed. The postmetacrista of *Nanocuris* does not have the postmetacrista cusp and does not form a carnassial notch. This feature is very similar to *Gurbanodelta*. The stylocone and parastyle in *Nanocuris* are fused together, forming the only dominant cusp along the stylar shelf border. The lower molar of *Nanocuris* has a large paraconid that is bigger and higher than the metaconid. A salient carnassial notch is developed between the paraconid and protoconid. The talonid of the lower molar in *Nanocuris* is relatively larger than that in *Gurbanodelta*. The talonid has a small hypoconid, hypoconulid and a cingulid-like entoconid, and the small talonid basin is enclosed by these three cusps. A strong cristid obliqua also is present in *Nanocuris*, and it extends mesially up to the tip of the metaconid. That feature is not present in other deltatheroidans.

The larger *Tsagandelta* is represented by a single a jaw fragment preserving m2 and part of the crown of m3 [6]. *Tsagandelta* has a paraconid that is larger than its metaconid, a sharp carnassial notch on the paracristid and a large mesiobuccal cuspid f. Those features are absent in *Gurbanodelta*. In addition, the talonid of *Tsagandelta* is relatively broader than that in *Gurbanodelta*, and the hypoconid and cristid obliqua are better developed than those in *Gurbanodelta*.

Phylogenetic Analysis

We added *Gurbanodelta kara* to the dataset of Luo et al. (2011, Morphobank Project X1599) [14] to examine the systematic position of *Gurbanodelta* to Deltatheroida within a broader sample of mammals, and the recent dataset of Rougier et al. (2015) [6] to examine the phylogenetic relationships between *Gurbanodelta* and other deltatheroidans. The strict consensus of the most parsimonious trees is in S-Figure 1

The m1 (IVPP V 22804) assigned to *Gurbanodelta kara* has a relatively small paraconid and relatively weakly-developed paracristid. These features are not "typical" for a deltatheroidan. When this lower molar of *Gurbanodelta kara* is not scored into the data matrix, the phylogenetic relationship between *Gurbanodelta* and other deltatheroidans remains unchanged (S-Figure 2, 3).

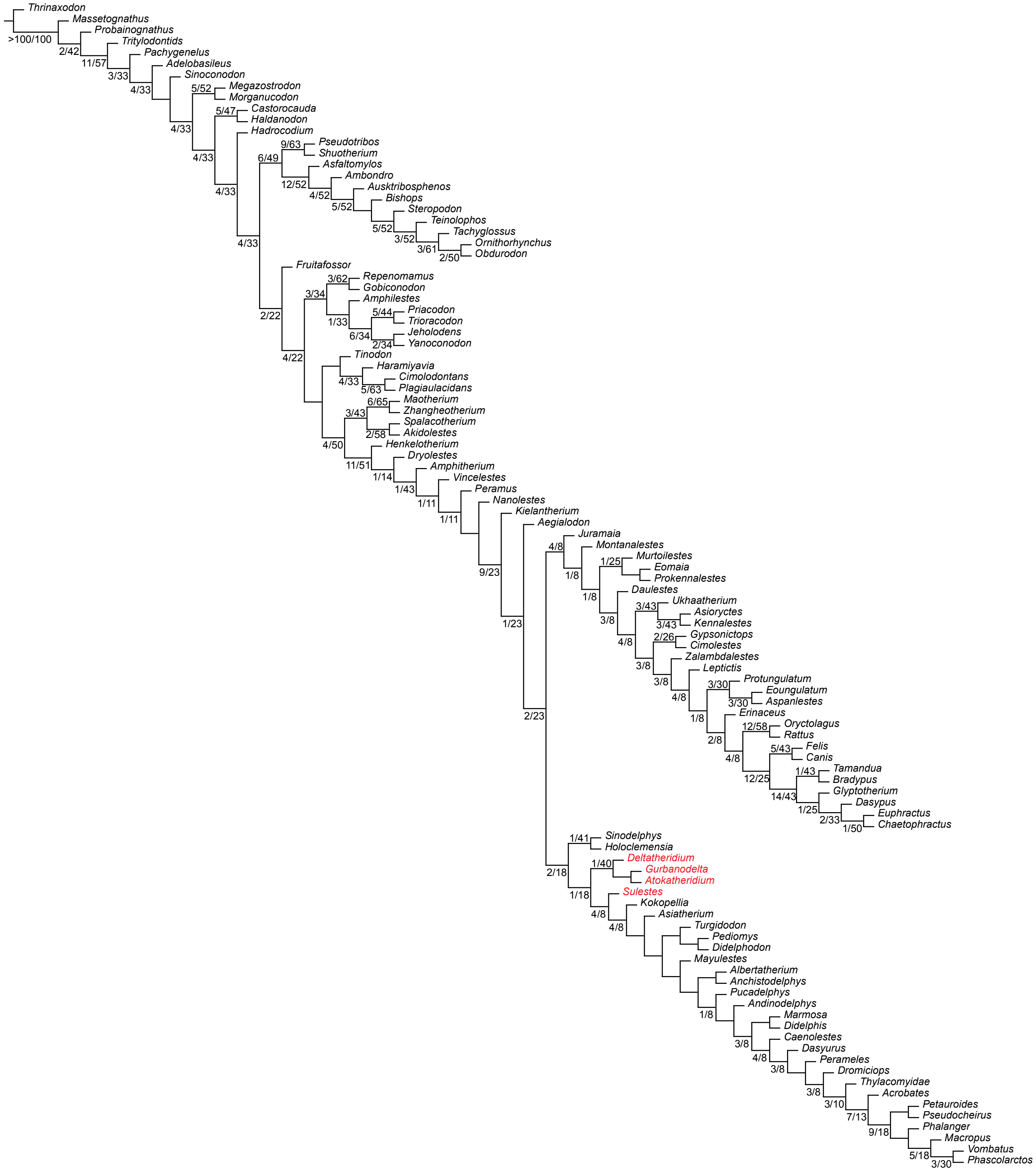

S-Figure 1. The strict consensus tree derived from 215 equally-parsimonious trees (2170 steps) resulting from the analysis of the dataset from Luo et al. (2011) [14]. The numbers before the slashes are the Bremer Support values; numbers after the slashes are Relative Supports [15,16]. Internodes without Bremer Support values indicate polytomies.Deltatheroidan taxa are indicated in red.

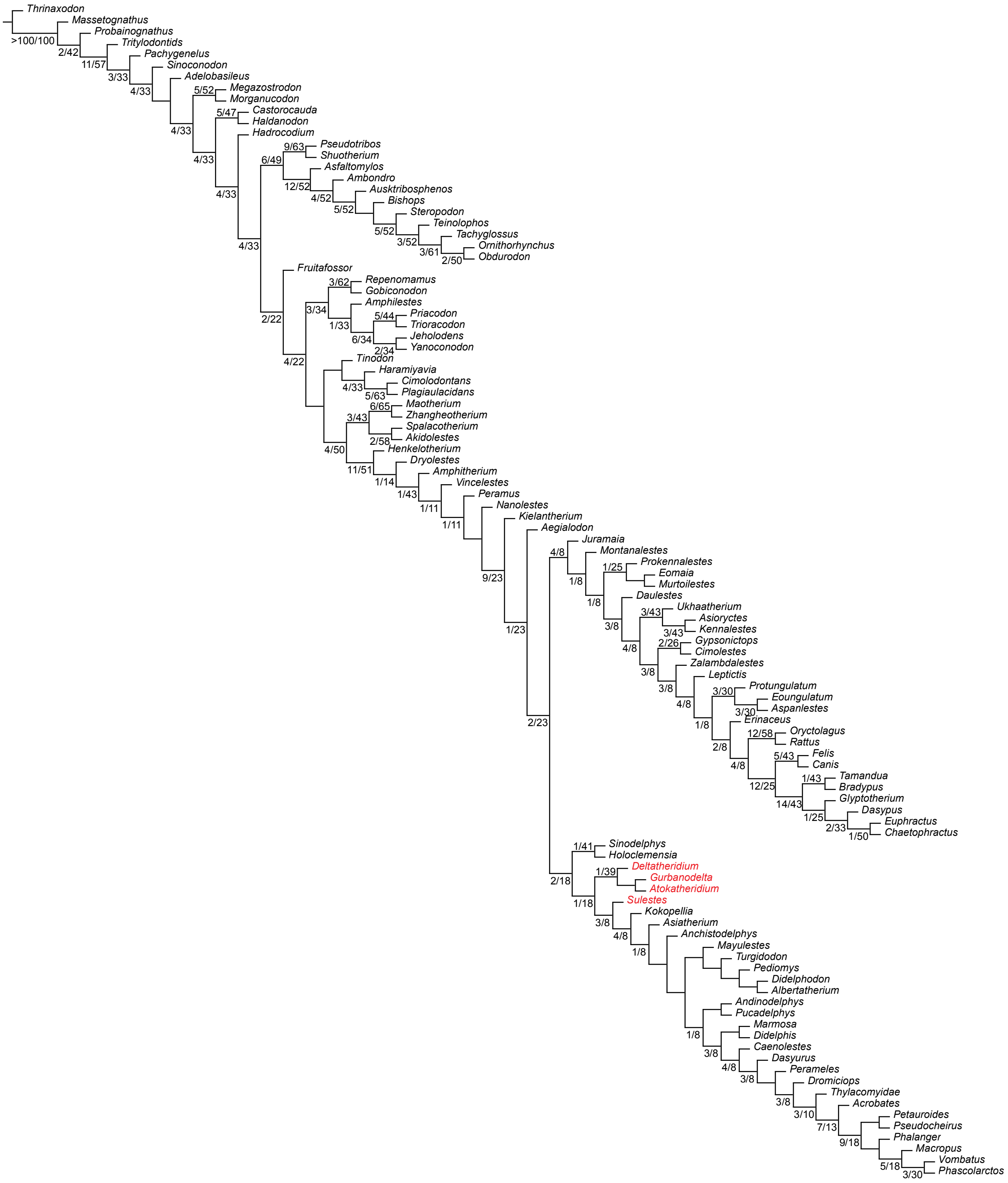

S-Figure 2. The strict consensus tree derived from 215 equally-parsimonious trees (2167 steps) resulting from the analysis of the dataset from Luo et al. (2011) [14]. The lower molar (IVPP V 22804) of *Gurbanodelta kara* is not included in the analysis. The numbers before the slashes are the Bremer Support values; numbers after the slashes are Relative Supports [15,16]. Internodes without Bremer Support values indicate polytomies. Delatheroidan taxa are indicated in red.

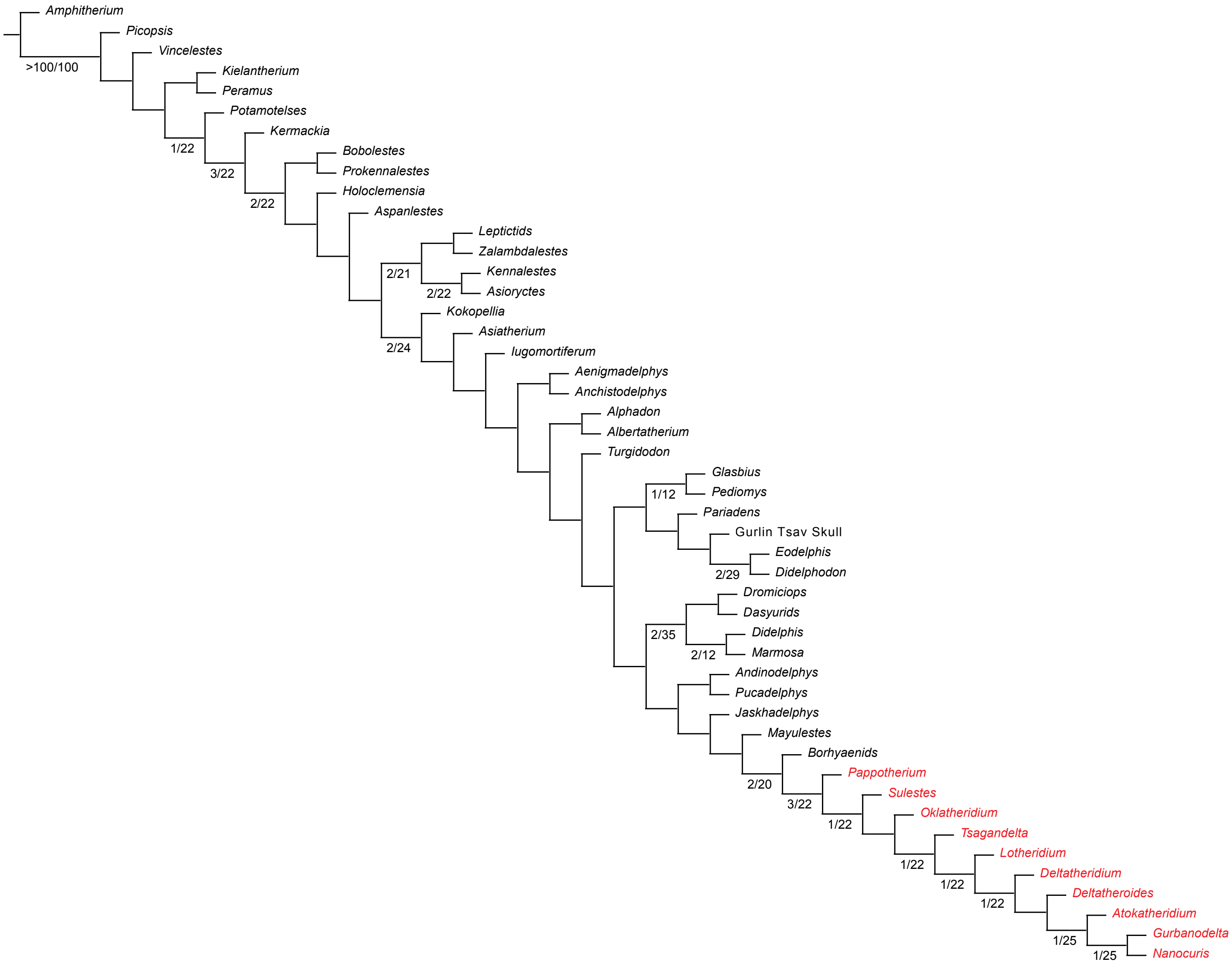

S-Figure 3. The strict consensus tree derived from 87 equally-parsimonious trees (560 steps) resulting from the analysis of the dataset from Rougier et al. (2015) [6]. The lower molar (IVPP V 22804) of *Gurbanodelta kara* is not included in the analysis. The numbers before the slashes are the Bremer Support values; numbers after the slashes are Relative Supports [15,16]. Internodes without Bremer Support values indicate polytomies. Deltatheroidan taxa are indicated in red.

References:

1 Kielan-Jaworowska, Z. & Cifelli, R. L. Primitive boreosphenidan mammal (? Deltatheroida) from the Early Cretaceous of Oklahoma. *Acta Palaeontologica Polonica* 46, 377-391 (2001).

2 Davis, B. M., Cifelli, R. L. & Kielan-Jaworowska, Z. in *Mammalian Evolutionary Morphology: A Tribute to Frederick S. Szalay* (eds Eric J. Sargis & Marian Dagosto) 3-24 (Springer, 2008).
3 Davis, B. M. & Cifelli, R. L. Reappraisal of the tribosphenidan mammals from the Trinity Group (Aptian–Albian) of Texas and Oklahoma. *Acta Palaeontologica Polonica* 56, 441-462 (2011).
4 Clemens, W. A., Lillegraven, J. A., Lindsay, E. H. & Simpson, G. G. in *Mesozoic Mammals: The First Two-thirds of Mammalian History* (eds J. A. Lillegraven, Z. Kielan-Jaworowska, & W. A. Clemens) 7-58 (University of California Press, 1979).
5 Rose, K. D. *The Beginning of the Age of Mammals*. 1-428 (The Johns Hopkins University Press, 2006).
6 Rougier, G. W., Davis, B. M. & Novacek, M. J. A deltatheroidan mammal from the Upper Cretaceous Baynshiree Formation, eastern Mongolia. *Cretaceous Research* 52, Part A, 167-177 (2015).
7 Wilson, G. P. & Riedel, J. A. New specimen reveals deltatheroidan affinities of the North American Late Cretaceous mammal *Nanocuris*. *J. Vert. Paleontol.* 30, 872-884 (2010).
8 Williamson, T. E., Brussatte, S. L. & Wilson, G. P. The origin and early evolution of metatherian mammals: the Cretaceous record. *Zookeys* 465, 1-76 (2014).
9 Averianov, A. O., Archibald, J. D. & Ekdale, E. G. New material of the Late Cretaceous deltatheroidan mammal *Sulestes* from Uzbekistan and phylogenetic reassessment of the metatherian-eutherian dichotomy. *Journal of Systematic Palaeontology* 8, 301 - 330 (2010).
10 Kielan-Jaworowska, Z., Cifelli, R. L. & Luo, Z. *Mammals from the Age of Dinosaurs: Structure, Relationships, and Paleobiology*. 1-630 (Columbia Univeristy Press, 2004).
11 Bi, S., Jin, X., Li, S. & Du, T. A new Cretaceous metatherian mammal from Henan, China. *PeerJ* 3, e896, doi:DOI:10.7717/peerj.896 (2015).
12 Rougier, G. W., Wible, J. R. & Novacek, M. J. Implications of *Deltatheridium* specimens for early marsupial history. *Nature* 396, 459-463 (1998).
13 Rougier, G. W., Wible, J. R. & Novacek, M. J. New specimen of *Deltatheroides cretacicus* (Metatheria, Deltatheroida) from the late Cretaceous of Mongolia. *Bulletin of Carnegie Museum of Natural History* 36, 245-266 (2004).
14 Luo, Z.-X., Yuan, C.-X., Meng, Q.-J. & Ji, Q. A Jurassic eutherian mammal and divergence of marsupials and placentals. *Nature* 476, 442-445 (2011).
15 Bremer, K. Branch support and tree stability. *Cladistics* 10, 295-304 (1994).
16 Goloboff, P. A. & Farris, J. S. Methods for quick consensus estimation. *Cladistics* 17, S26-S34 (2001).
